# Putrescine mitigates NaCl-induced stress by modulating gene expression, antioxidants, and ethylene level in tomato

**DOI:** 10.1080/15592324.2025.2515431

**Published:** 2025-06-16

**Authors:** Yalaga Rama Rao, Priya Yadav, Varsha Rani, Devayani Muley, Ranjan Kumar Sahoo, Brajendra, Shashi Bhushan Kumar, Ritu Gill, Sarvajeet Singh Gill, Mohammad Wahid Ansari, Narendra Tuteja

**Affiliations:** a Department of Biotechnology, Vignan’s Foundation for Science, Technology & Research, Guntur, India; b Department of Botany, Zakir Husain Delhi College, University of Delhi, New Delhi, India; c Department of Crop Physiology, Birsa Agricultural University, Kanke, Ranchi, Jharkhand, India; d Department of Biotechnology, Centurion University of Technology and Management, Bhubaneswar, India; e Division of Soil Science, ICAR Indian Institute of Rice Research, Hyderabad, India; f Stress Physiology and Molecular Biology Lab, Centre for Biotechnology, MD University, Rohtak, India; g Plant Molecular Biology Group, International Centre for Genetic Engineering and Biotechnology, New Delhi, India

**Keywords:** Antioxidant enzymes, oxidative stress, putrescine, salinity stress, stress alleviation, tomato

## Abstract

Plant development and productivity are significantly hindered by salt stress, leading to substantial financial losses in the agriculture sector. Salinity stress negatively impacts the overall growth, physiology, and metabolism of plants. Specifically, NaCl stress is particularly harmful to tomato plants, causing suppression of seedling growth, accumulation of sodium (Na^+^) and chloride (Cl^−^) ions, disrupted ion homeostasis, reduced proline and chlorophyll content, and impairment of antioxidant enzyme systems. This research aimed to investigate the role of exogenous putrescine (PUT) application on tomato (*Solanum lycopersicum* L.) seedlings under NaCl stress (250 mm) to determine its potential protective effects. Various physio-biochemical attributes were estimated using precise protocols for NaCl-treated, PUT-treated, and untreated controlled tomato seedlings also analyzed for the expression of ACS1, NHX1, HKT1;2, and SOS1 genes. Additionally, ACC synthase activity, ethylene content, electrolyte leakage, proline content, Na^+^ and potassium (K^+^) ion content, lycopene content, and antioxidant enzyme activities were examined. Results indicated that PUT application enhanced the expression of ACS1, NHX1, HKT1;2, and SOS1 genes increase the ACC synthase activity, ethylene content, proline content, and Na^+^ and K^+^ ion content, while reducing electrolyte leakage. Furthermore, PUT application significantly increased the activity of superoxide dismutase (SOD), catalase (CAT), ascorbate peroxidase (APX), and glutathione reductase (GR), as well as other morphological parameters. Overall, our research demonstrated the potential benefits of PUT applications for enhancing crop growth and improving salt stress tolerance, which are crucial for agronomy.

## 1. Introduction

Loss of cultivable land due to soil salinization is one of the major challenges faced by world in order to fulfil the dietary needs of fast-growing population within limited resources.^1,2^ Salinity stress is one of the major abiotic stresses that impart major negative/adverse effect on plants right from seed germination till maturity and fruit or seed production.^3^ Plant growth suppression by salinity has been explained by two theories: either ion toxicity or osmotic disturbance.^4^ According to reports, salinity inhibits various physiological processes, such as the intake of nutrients by disturbing nutrition channels, ion imbalance that results in ion toxicity, the pace at which photosynthetic activity occurs, and plants osmosis by disturbing absorption of water.^5,6^ Plants under salinity stress not only experience disturbance in photosynthetic pigments and chlorophyll production whereas, production and flux of secondary metabolites (such as polyphenolics, flavonoids, saponins, anthocyanins and tannins) also changes, which disrupt physiological, morphological and metabolic processes (chlorophyll content, seed germination and seedling growth, reduced shoot and root length, fresh and dry weight).^7–9^ Additionally, it is shown that plants under salt stress exhibit distinct protein and carbohydrate levels. There are numerous factors, such as plant species, developmental phases, the severity and duration of the stress itself, that influence these positive or negative metabolomic changes under salinity stress.^10^ Tomato (*Solanum lycopersicum* L.) member of Solanaceae family is a major crop that is grown worldwide both under protected culture and in open fields.^11^ Due to its many applications and presence of plenty of nutritious and antioxidant bioactive substances, it is among the most significant and popular crop for horticulture.^12^ The significant amounts of antioxidants included in tomato fruits, such as lycopene pigment, vitamins like C, and numerous minerals, can slow the course of a number of serious human diseases, including breast, colon, and prostate cancer.^13^ Whereas, abiotic stresses and conventional farming practices also significantly affect the yield of tomatoes and the quality of the fruit.^14^ A wide range of tomato cultivars used for commercial purposes exhibit varying degrees of sensitivity to salt stress. The two main approaches to managing salinity stress are developing genotypes resistant to unfavorable environments or developing a new watering method. Numerous earlier methods, used in order to lessen the negative effects of salinity are use of compost and vermicompost,^15^ application of plant growth-promoting bacteria,^16^ foliar application of indole-3-acetic acid,^17^ and root and foliar application of salicylic acid (SA).^18^ There are few reports regarding exogenous application of polyamines (PAs) for salinity stress mitigation.^19^ The predominant PAs found in higher plants are diamine Put, triamine spermidine (Spd), tetramine spermine (Spm), thermospermine (Tspm), and cadaverine (Cad).^20–23^ PUT is the abundant among all and it has potential being a central product of the PA biosynthetic pathway.^19^ PUT is also one of the emerging metabolites involved in the growth of plant, their stress tolerance, and stress-response mechanisms.^24^ PUT improves salt tolerance by controlling, ASA-GSH cycle, efficiency of photosynthetic system, and ion homeostasis. In case of strawberries PUT cause a notable change in antioxidants activity, protein, carbohydrate amount and lycopene content. Also, it found helpful in maintaining osmolyte homeostasis and increased DPPH.^25^ Combined treatments of PUT with other components also found beneficial as in rice PUT along with PGPR (strain JIV1) helps in mitigating salt stress and improve plants growth by enhancing photosynthetic efficiency also enhance activity of urease and protease enzymes by a significant fold.^26^ Through increased photosynthesis, antioxidant capability, decreased Na^+^ buildup, and increased K^+^ content, exogenous spermidine (Spd) application successfully reduced the salinity damage experienced by grapevines.^27^ Exogenous application of spermine (Spm) and spermidine (Spd) in *Brassica napus* L. significantly elevates various physiological aspects of salinity stressed plants. It also enhances the expression of antioxidant enzyme (SOD, CAT, GR, and DHAR) genes, genes of polyamine pathways, and genes of enzymes of Calvin cycle in order to mitigate salinity stress.^28^ Nonetheless, numerous facets of tolerance for salt stress mediated by exogenous PUT in tomatoes are yet unclear. Given this context, the current study was conducted to examine the probable functions and underlying mechanisms of tomato salt-stress tolerance mediated by PUT. To investigate the mechanism of salt-stress alleviation by PUT application, various morphological, physiological, biochemical, and molecular factors that influence salinity tolerance were assessed.

## 2. Methods and material

### 2.1. Plant material and growth conditions

Seeds of tomato (*Solanum lycopersicum* L. cv. Pusa Ruby) were procured from IARI (Indian Agricultural Research Institute), New Delhi, India. The experiments were carried out in the greenhouses of the International Centre for Genetic Engineering and Biotechnology (ICGEB), New Delhi, India. The plants were grown in earthen pots at 28°C, 16 h light at 100–125 μmol/m^2^/s^1^ and 70–75% relative humidity. Surface sterilization of seeds was done using 0.01% HgCl_2_ and later washed by distilled water 2–3 times. Seeds were sown in pots filled with garden soil, sand, and farm manure (3:1:1), and irrigation was done whenever required using tap water. Treatment of control, 250 mm NaCl, and 250 mm NaCl plus 1000 µM PUT was given to plants after 30 days for three consecutive days. The plants were given full development conditions and evaluated for several physiological and growth parameters after 45 days of sowing.

### 2.2. Isolation of RNA transcripts for qRT-PCR (qRT-PCR)

Gene sequences have been obtained from the NCBI (National Centre for Biotechnology Information) sequencing database using the websites with corresponding accession number (https://www.ncbi.nlm.nih.gov/nuccore/AJ306630.1;https://www.ncbi.nlm.nih.gov/nuccore/NM_001302904.1;https://www.ncbi.nlm.nih.gov/nuccore/NM_001247249.3;https://www.ncbi.nlm.nih.gov/nuccore/NM_001247769.3).NHX1., HKT1–2, ACS1, and SOS1 genes were selected for expression study, and their sequences were isolated. The accession number of the HKT 1;2 gene is NM_001302904. This gene is useful to increase the K^+^ ions in the xylem vessels. The accession number of NHX1 gene is AJ306630. This gene is helpful for removing the excess Na^2+^ for diminishing the salinity stress in tomato plants. The accession number of the SOS1 gene is NM −001247769 which is an antiporter gene for excluding the Na^2+^ from the cell to the apoplast area. Finally, the accession number of the ACS 2 gene is NM_001247249. This gene is responsible for the production of ethylene by regulating ACS enzyme gene expression. Samples were taken from tomato plant leaves that were treated with control, 250 mm NaCl, 250 mm NaCl plus 1000µM PUT for qRT-PCR. According to the manufacturer’s instructions, Poly-A ribonucleic acids are separated using the TRIzol Reagent (Invitrogen, http://www.invitrogen.com).^29^ cDNA was constructed using Reverse H aid minus set, a cDNA amplification kit.^30^ The relative levels of expression of these genes in treated and controlled plants are analyzed through qRT-PCR by following the protocol of.^31^ The levels of gene expression associated with HKT1;2, NHX1, ACS2, and SOS1 were determined in the untreated and treated plant using the 2-ΔΔCt method.^32,33^

### 2.3. ACS activity assessment and quantification of ethylene content

#### 2.3.1. Extraction of ACC synthase and activity assessment

Using the methodology established by Kato et al.^34^ the ACS enzyme was assessed. The 500 mg of tissue obtained from control, 250 mm NaCl, and 250 mm NaCl plus 1000 μM PUT-treated plants, were used to make a uniform mixture using pestle and mortar in 3 ml extraction buffer which contains buffer solution (0.1 M EPPS-KOH) by maintaining the pH value 8.5, mercaptoethanol (10 mm) followed by pyridoxal phosphate (10 μM) at the temperature of 4°C. The mixture was centrifuged for 20 min at 14,000 rpm and 4^º^C. Afterward, the supernatant was desalted, and it was passed through a column (PD-10; disposable columns containing Sephadex medium) containing 10 mm (EPPS-KOH buffer) at pH 8.5, which contained 10 mm 2-mercaptoethanol and 10 μM pyridoxal phosphate. Further, the ACC synthase activity was estimated using the extract prepared, later (50 μM) of SAM was added and the extraction of the enzyme prepared out of 1 ml volume. The entire mixture of the reaction was incubated at a temperature of 30^º^C for 30 min. Then, the reaction was terminated with the addition of HgCl_2_ (0.1 ml of 40 mm). ACC produced in the reaction mixture was investigated by converting it to ethylene using the Lizada and Yang procedure. ^35^ Protein content was assessed with the help of the protocol framed by Lowry.^36^ The current ACC synthase concentration was computed as nano mol mg protein^−1^ h^−1^.

#### 2.3.2. Quantification of ethylene content

The ethylene content of tissue extracted from leaves at a concentration of control, 250 mm NaCl, and 250 mm NaCl plus 1000 μM PUT was determined using the Nakatsuka process.^37^ Gas chromatography was used to quantify the ethylene content of the sample under an identical set of factors as recommended by Ansari et al.^28^ The ethylene quantification method involved placing plant tissues in a vacuum sealed system to extract intercellular gases. Tissues were immersed in a saturated ammonium sulfate solution to minimize ethylene solubility. A surfactant (0.01% Tween 20) helped remove air bubbles from the tissue surface. After preparing the tissue, a constant vacuum was applied for 2 min, causing internal gases to expand and collect in a flask. The gas sample was then analyzed using gas chromatography. Calibration was performed using a known 100 ppm ethylene standard.

### 2.4. Determination of electrolyte leakage

The tomato plants that are collected from control, 250 mm NaCl, and 250 mm NaCl plus 1000 μM PUT were immediately washed thoroughly by using distilled water in order to remove the sticky ions entered due to salinity treatment. Then, small ruptures were made on the surface of the treated and control plants. The method established by Bajji was employed to ascertain the electrolyte leakage.^38^ The entire inorganic ions released were evaluated by following the protocol developed by Sullivan and Ross.^39^ Twenty leaf discs were taken that were boiling and contain 10 ml of water that was deionized and EC was calculated (ECa). The electrical conductivity (ECb) is being calculated by exposing the contents to heat at temperatures ranging from 45ºC to 55ºC for about 30 min in water baths, and the electron conductivity was measured. After that, the (EC’c) was measured by boiling the content at 100ºC for 10 min. The leakage of electrolytes is being calculated with the help of the following formula.

$$\mathrm{EL}\;\left(\%\right)\;=\left[\left(\mathrm{ECb}-\mathrm{ECa}\right)/\left(\mathrm{ECc}\right)\right]\;\times 100$$

### 2.5. Estimation of proline content

Proline estimation was done by following the method designed by Bates et al.^40^ 100 mg of the leaf sample has been taken from plants which were exposed to 250 mm NaCl, 250 mm NaCl plus 1000 µM PUT, and control. Subsequently, the material was extracted using 3% sulfosalicylic acid. Following that, glacial acetic acid (2 ml) and ninhydrin (2 ml) were added in equal amounts. The sample was exposed to heat at a temperature of 100°C. A 5 ml toluene solution was then added and allowed to cool. A spectrophotometer was used to determine the absorbance at 528 nm. By using the pure proline, a standard graph was formed and used to find absorbance for the toluene layer.

### 2.6. Evaluation of Na^+^ and K^+^ ions and lycopene content

100 mg of leaf tissue was taken from control, 250 mm NaCl, and the plants treated with NaCl plus 1000 µM PUT to quantify Na^+^ and K^+^ ions. The 0.1% nitric acid was used to degrade the tissue, and distilled water was then used to extract the ions. Further, the extraction was brought to a boil twice for half an hour. A flame photometer was utilized to assess the concentrations of Na^+^ and K^+^ ions in the filtered extract.^41^ To measure the lycopene content, 100 mg of leaf tissue was homogenized in 50 mL phosphate buffer of pH 6.8 using a mortar and pestle. Filter it using muslin cloth. Then, in 2 mL filtration the 8.0 mL of a hexane:acetone:ethanol (2:1:1) was added. The samples were mixed properly, and the absorbance was recorded at 503 nm.

### 2.7. Estimation of activity of antioxidant enzymes

The fresh leaves of the tomato plant collected from 250 mm NaCl, 250 mm NaCl plus 1000 µM PUT, and control were homogenized in 50 mm PBS (phosphate buffer solution) pH 7.0. Centrifugation of the homogenized mixture was performed for approximately 10 min at 4ºC. Following this, the supernatant that had been collected was utilized to assess the antioxidant enzymes CAT, SOD, APX, and GR. The Chance and Maehly^42^ procedure was used in order to estimate APX (E.C. 1.11.1.7). The extraction of enzyme (0.1 ml) was added to the mixture of a reaction that consisted of phosphate buffer (pH 6.8) and pyrogallol along with hydrogen peroxide (1%). The evaluation of the enzyme extract was based on the absorbance change for every 20 (ELICO SL171 minI SPEC) seconds for about 2 min at 420 nm spectrophotometry. A control set was also made by the addition of DDW instead of the extraction enzyme CAT, which was estimated by adding the enzyme extract (1.0 ml) to the reaction mixture that contains phosphate buffer and H_2_O (0.1 m) (pH 6.8). H_2_SO_4_ was added to the reaction mixture. Later, this was incubated for a minute at a temperature of 25ºC followed by the process of titration against potassium permanganate solution.^42^

The capacity to photochemically impede the reduction of nitro blue tetrazolium was determined in order to estimate SOD (E.C. 1.15.1.1) using the Beauchamp and Fridovich technique.^43^ The method of Foyer and Halliwell^44^ was followed for the purpose of determining GR. The procedure contains phosphate buffer (25 mm) pH7.8, oxidized glutathione (0.5 mm), NADPH (0.2 mm) along with the extraction of the enzyme GR was estimated by observing oxidation of NADH that depends up on glutathione at 340 nm.

### 2.8. Determination of morphological parameters

Ruler was used to measure the length of the roots and shoots in control, 250 mm NaCl, and 250 mm NaCl plus 1000 µM PUT-treated tomato plants. The number of leaves and leaf area in 250 mm NaCl, 250 mm NaCl plus 1000 µM PUT-treated and control plants was computed.^45,46^ Using a weighing machine, the fresh weight of the roots and shoots in 250 mm NaCl, 250 mm NaCl plus 1000 µM PUT-treated and control plants were measured, respectively.^45^

### 2.9. Statistical analysis

We followed the randomized block design for the experiments. Each treatment was considered to have three replicates (*n* = 3). Standard errors were computed after a statistical analysis of the collected data. The average values derived from control, 250 mm NaCl, and 250 mm NaCl plus 1000 µM PUT-treated tomato plants were determined using One-Way ANOVA (ANALYSIS OF VARIANCE) and SPSS 10.0. The significant data at *p* < 0.05 were used to calculate the least significant differences or LSDs.

## 3. Results

### 3.1. Gene expression of ACS2, SOS1, NHX1, HKT1;2 in treated and untreated plants

**Figure 1.**
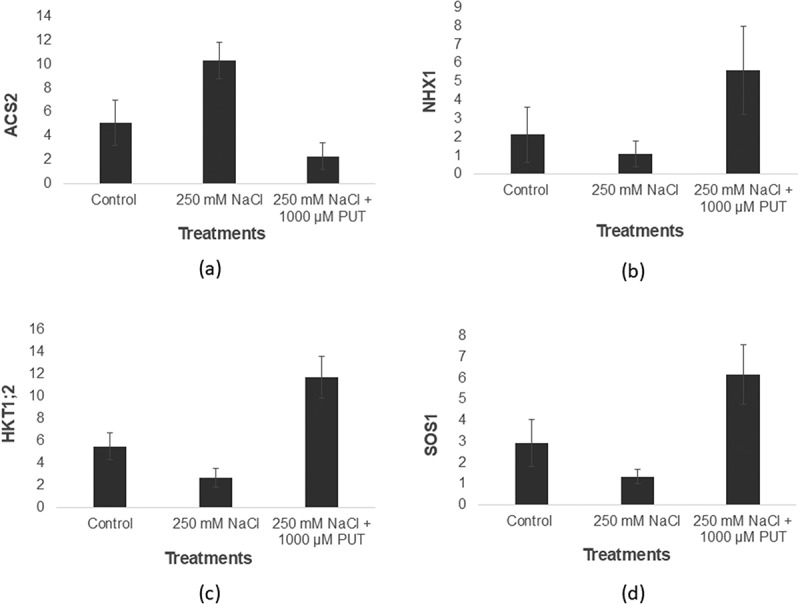
Response of tomato plants to salinity stress (250 mm NaCl) and 250 mm NaCl plus 1000 μM PUT as compared to control. The endogenous transcript levels of *ACS2*, *NHX1*, *SOS1*, and *HKT1;2* (a–d), in treated and untreated tomato plants compared to control.

When tomato plants were exposed to 250 mm NaCl, they experienced severe salt stress, which negatively impacted their performance. However, tomato plant growth was improved under 1000 µM PUT treatment as compared to salinity stress treatment. Expression of ACS2 was found to be decreased in the case of 250 mm NaCl plus 1000 µM PUT-treated tomato plants (2.3 folds) whereas salt affected by 250 mm NaCl plant showed increased expression (10.3 folds) and in the control expression was 5.1 folds (Figure 1(a)). Conversely, there was a notable improvement in the NHX1 transcripts under 250 mm NaCl plus 1000 µM PUT-treated tomato plants (5.61 folds), whereas 250 mm NaCl-treated showed (1.1 folds) and 0 mm NaCl (H_2_O) showed (2.13 folds) expression (Figure 1(b)). Likewise, after treating tomato plants with salt, a base-level increase of SOS1 transcripts was seen, whereas substantial amount of expression was seen in the case of 250 mm NaCl plus 1000 µM PUT-treated plants, respectively (1.34 and 6.17 folds), and control showed a 2.92-fold increase (Figure 1c). Similarly, there was a notable rise in HKT1;2 transcript abundance in plants with 250 mm NaCl plus 1000 µM PUT-treated (11.75 folds) with respect to 250 mm NaCl and control, respectively (2.66 and 5.51 folds) (Figure 1(d)).

### 3.2. Estimation of activity of ACS and determination of ethylene content

**Figure 2.**
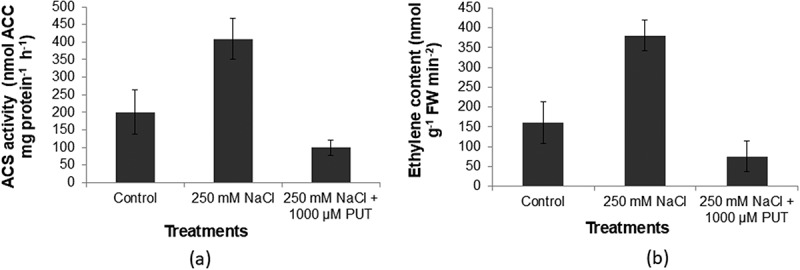
Effect of salinity stress (250 mm NaCl) and 250 mm NaCl plus 1000 μM PUT on ACC synthase enzyme activity (pmol ACC mg protein^− 1^h^− 1^) (a) and ethylene content (pmol g^− 1^ FW min ^− 2^) (b) in treated and untreated compared to control.

The current investigation indicated that the 250 mm NaCl-treated sample had the highest ACS activity (380.9 nanomole mg p^−1^ h^−1^) whereas in the case of untreated control (161.3 nanomole mg p^−1^ h^−1^). A decrease of 2.1 folds in the ACS enzyme activity levels was observed in 250 mm NaCl plus 1000 µM PUT-treated plants (75.5 nanomole mg p^−1^ h^−1^) (Figure 2(a)). A comparable pattern was observed for the internal ethylene concentration, which was notably low for 250 mm NaCl plus 1000 µM PUT-treated plants (99.7 mg p^−1^ h^−1^) compared to control, and salt-treated for 250 mm NaCl (200.6 and 408.2 mg p^−1^ h^−1^) (Figure 2(b)).

### 3.3. Determination of electrolyte leakage

**Figure 3.**
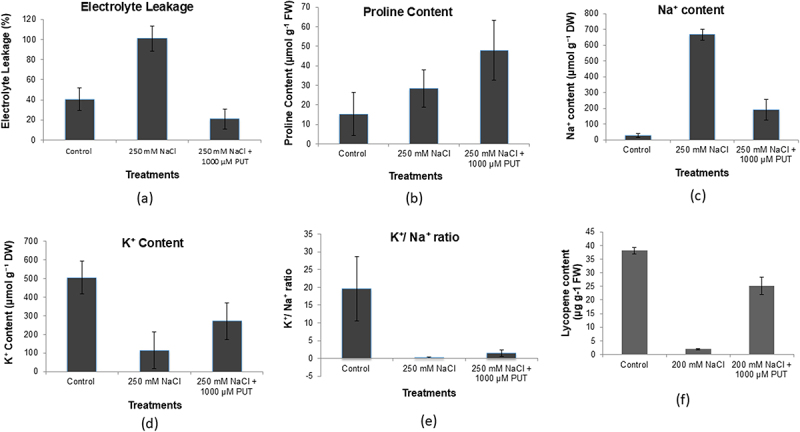
Effect of salinity stress (250 mm NaCl) and 250 mm NaCl plus 1000 μM PUT on electrolyte leakage (a), endogenous proline content (b), endogenous Na^+^ content (c), endogenous K^+^ content (d) and ratio of K^+^/Na^+^ (e) and lycopene content (f) in treated compared to control plants.

The electrolyte leakage (%) was found to be induced in 250 mm NaCl. Whereas in the case of control and 250 mm NaCl plus 1000 µM PUT-treated plants the electrolyte levels were reduced by 2.5 folds and 4.9 folds, respectively. The least electrolyte leakage was observed in PUT-treated plants (Figure 3(a)). The lycopene (µg g^−1^FW) content was significantly higher in 250 mm NaCl plus 1000 μM PUT as compared

### 3.4. Quantification of proline content

There was an increase in the proline content (μmol/M g^−1^ FW) in 250 mm NaCl-treated (28.4 μmol g^−1^ FW) increase is about 2 folds and in 250 mm NaCl plus 1000 μMPUT (47.9 μmol g^−1^ FW) treated tomato increase is 3.3 folds as compared to control (15.2 μmol g^−1^ FW) (Figure 3(b)).

### 3.5. Assessment of Na^+^ and K^+^ ions and lycopene content

Highest Na^+^ ions were observed in 250 mm NaCl-treated plants 666.7 μmol p DW^−1^ 20 folds more than the control followed by 250 mm NaCl plus 1000 μM PUT-treated plants 189.7 μmol p DW^−1^ 3 folds more compared to control and control plants showing the least amount of Na^+^ ions (Figure 3c). It was noted that the K^+^ ion concentrations were significantly lowered by 5 folds in the plant under salt stress with a value of 114.6 μmol p DW^−1^, whereas, 250 mm NaCl plus 1000 μM PUT-treated plants showed 2.5 folds decrease compared to control and control showed a high amount of K^+^ ions 503.9 μmol p DW^−1^ (Figure 3(d)). The K^+^/Na^+^ ion levels were lowered by 18 folds in salt-treated plants. In PUT-treated plants, a decrease of 3.6 folds was observed, whereas in control the ratio was recorded highest (Figure 3(e)). The lycopene content (µg g^−1^ FW) was significantly higher in 250 mm NaCl plus 1000 μM PUT-treated plants as compared to 250 mm NaCl-treated plants. Its endogenous content was significantly reduced under 250 mm NaCl stress as compared to the control plant (Figure 3(f)).

### 3.6. Antioxidant enzymes activity

**Figure 4.**
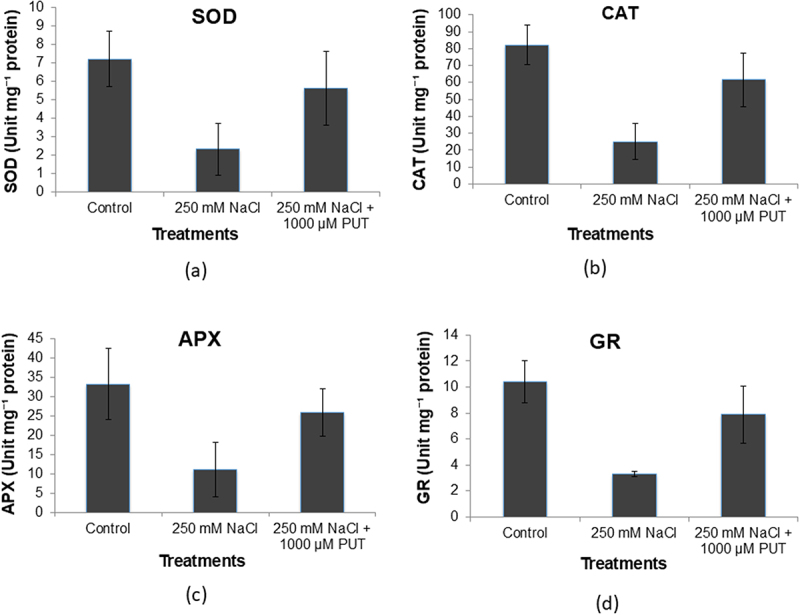
Effect of salinity stress (250 mm NaCl) and 250 mm NaCl plus 1000 μM PUT on antioxidants enzymes SOD (a), CAT (b), APX (c) and GR (d) compared to control.

The results of the antioxidant assay indicate that the plants that were treated with 250 mm NaCl plus 1000 μM PUT and control showed better SOD levels 5.6 unit mg^−1^ and 7.2 unit mg^−1^ protein approximately that was 1.2 folds and 3.1 folds higher, respectively, compared to salt-treated. Conversely, plants that underwent treatment with 250 mm NaCl showed a lower SOD content 2.3 unit mg^−1^ protein (Figure 4(a)). Whereas, enzyme activity levels of CAT were higher by 1.3 folds and 3 folds when plants were treated with 250 mm NaCl plus 1000 μM PUT and under control 61.5 and 82.2 unit mg^−1^ protein, respectively, compared to 250mM NaCl-treated were 25.1 unit mg^−1^ protein was observed (Figure 4(b)). It was observed in the instance of APX that plants treated with 250 mm NaCl plus 1000 μM PUT showed 1.2 folds more activity compared with the 250 mm NaCl-treated plant (Figure 4(c)). GR has also shown a comparable tendency (Figure 4(d)) high values of antioxidant enzymes recorded in the plants exposed to PUT compared to salt-treated.

### 3.7. Morphological parameters

**Figure 5.**
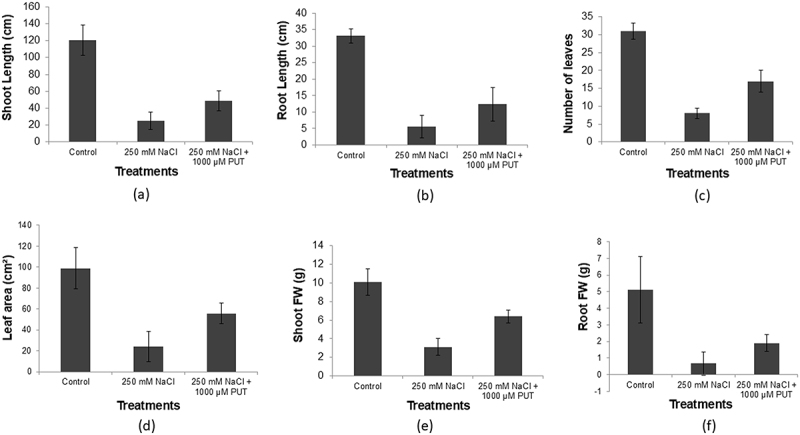
Effect of salinity stress (250 mm NaCl) and 250 mm NaCl plus 1000 μM PUT on shoot length (a), root length (b), number of leaves (c), leaf area per plant (d), shoot fresh weight (e) and root fresh weight (g) compared to control.

Like other parameters, morphological features are also affected by salt stress. The PUT treatments restore stress-inducing influence and strengthen the development factors and morphological parameters associated with the exterior characteristics of the plant, enabling it to withstand a higher level of salt. In 250 mm NaCl plus 1000 μM PUT-treated plants, the shoot length was found to be high when compared with the 250 mm NaCl-treated plants (48.7, and 25.1 cm respectively), whereas, control showed largest shoot length (120.2 cm) (Figure 5(a)). Comparable patterns have been observed in their root lengths in plants treated with 250 mm NaCl, 250 mm NaCl plus 1000 μM PUT, and control (5.6, 12.4, and 33.1 respectively) (Figure 5(b)). Similarly, 1000 μM PUT treatment substantially increased the number of leaves (17, 8, and 31) and leaf surface area (55.7, 24.3 and 99 cm^2^) compared to salt-treated, whereas in the case of control it was higher than the other two, respectively (Figure 5(c,d)). The similar trend was noticed for fresh weight of shoots (6.4, 3.1, and 10.1 g) among the plants treated with 250 mm NaCl plus 1000 μM PUT (Figure 5(d)) and fresh weight of roots (1.9, 0.67, and 5.1 g) (Figure 5(f)) in comparison, salt affected, whereas control plants reported with highest number, respectively.

## 4. Discussion

**Figure 6.**
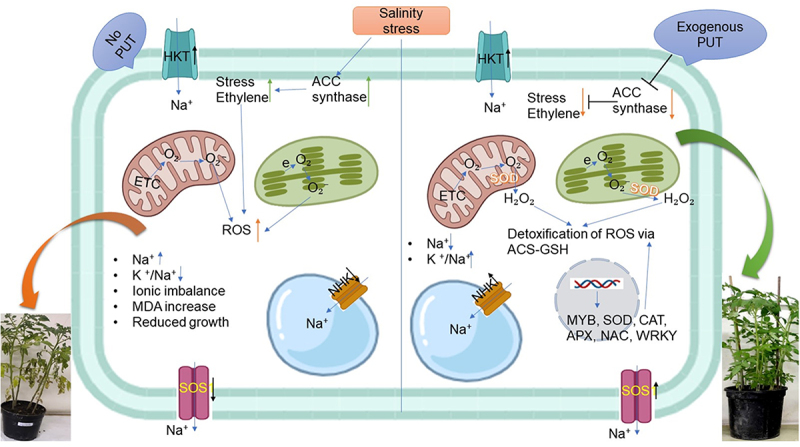
Schematic diagram of salinity stress tolerance mechanisms mediated through PUT to regulate ACS, NHX, SOS1 and HKT1;2 genes expression, ion balance, and improved antioxidants enzymes activity. ROS, reactive oxygen species; SOD, superoxide dismutase; etc electron transport chain; MDA, malondialdehyde; HKT, high-affinity potassium transporter; NHK, sodium-hydrogen exchanger; SOS, salt overly-sensitive.

A significant environmental hazard that keeps crops from reaching their full genetic potential is salinity, which poses multiple growth and developmental restrictions.^2,3,9^ Increased salt stress, on the other hand, caused Na^+^ buildup, reduced plant development, and enhanced oxidative damage such as electrical leakage and peroxidation of lipids. ^47^ Exogenous Put is essential for reducing the adverse effects of salt exposure and boosting pea plants’ ability to withstand salinity since it dramatically improves germination and seedling growth. ^48^ PUT treatment of chickpea seedlings produces changes in biochemical and enzymatical parameters, and their ability to withstand stress was determined to help them overcome problems associated with stress.^49^ Thus, one practical way to boost crop output is to choose and enhance genotypes that are resistant to salt.^50^ Moreover, a deeper comprehension of tolerance processes and the identification of significant and trustworthy physiological and biochemical indicators linked to salt tolerance are needed.^51,52^ Numerous physiological and molecular investigations in plants have demonstrated the importance of free PAs in controlling abiotic stress tolerance responses (Figure 6).^19,53^ According to research on rice seedlings, PAs can modify cell membrane integrity during salinity stress since they are ROS scavengers and antioxidants.^19,54^ In the current research, we assessed how exogenous PUT administration influences and facilitates the initiation of defense mechanisms to lessen the detrimental effects of salt stress on plant growth, shoot length, root length, fresh shoot and root weight, and number of leaves of tomato seedlings. This work offers a fresh physiological foundation for additional investigation into the PUT-mediated salt stress tolerance regulating mechanism in tomatoes and points to a possible link between stress ethylene production and salinity stress mitigation. In the present study, expression of various transport genes (NHX1, HKT1;2, and SOS1) was found to be enhanced on PUT application under NaCl (250 mm) treatment (Figure 1). The efficiency with which plants absorb and utilize ions is enhanced by membrane-embedded transporter proteins. The Na^+^/H^+^ transporters encoded by NHX genes are members of the cation-proton antiporter 1 family. NHXs have significant functions in maintaining Na^+^ and K^+^ homeostasis.^55^ The most effective method for maintaining ionic homeostasis in plants under salt stress is provided by the membrane and vacuolar Na^+^/H^+^ antiporters. The ability of plants to withstand salinity stress has been enhanced through the characterization and expression of vacuolar NHX antiporters in heterologous systems.^56^ Collectively implies that NHX1 ameliorates the vacuole’s Na^+^ sequestration and the root cell’s cytosolic and vacuolar K^+^ retention, so providing relief from salt stress.^57^ The majority of vascular bundles/tissue, xylem, phloem, root cortex, and epidermis express HKT transporters.^58,59^ Na^+^ is excluded by HKT transporters from the leaf xylem sap, preventing Na^+^ from penetrating the shoots and harming the photosynthetic cells. Using genetic engineering and traditional plant breeding techniques, modification of the expression of the HKT gene has been shown to be an effective strategy for increasing the salinity tolerance of economically significant crop plants.^60,61^ Na^+^ homeostasis is facilitated by Na^+^/H^+^ exchanger SOS1, which extrudes the ion from root epidermal cells. Research findings of the Na^+^ root/shoot division by SOS1 under different salinity conditions indicate that SOS1 has a role in the redistribution of Na^+^ between the root and shoot, presumably in association with HKT1;2. SOS1 is expressed more frequently in xylem parenchyma cells.^62,63^

**Figure 7.**
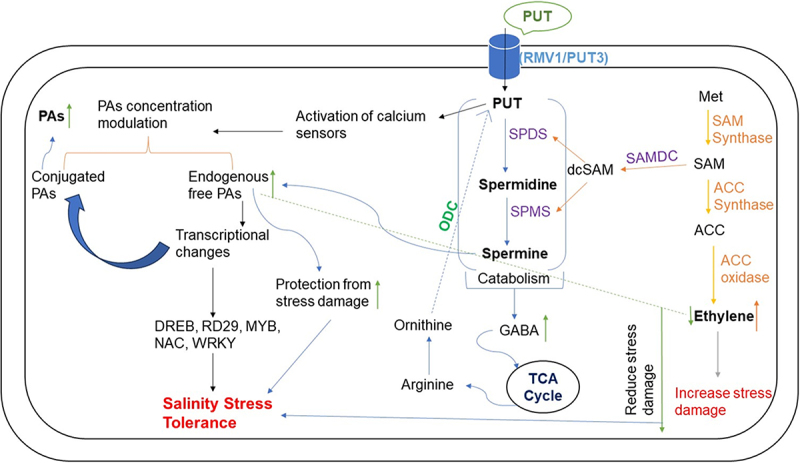
An illustration of the intricate relationship between polyamines and plant’s reaction to salinity stress. Exogenous Put application activate calcium sensor that results in downstream signaling activation and endogenous polyamines synthesis pathway activation. Further decreased the ethylene production and helps in stress tolerance. PUT, putrescine; ODC, ornithine decarboxylase; SPMS, spermine synthase; SPDS, spermidine synthase; SAMS, S-adenosylmethionine synthase; ACO, 1-aminocyclopropane-1-carboxylate oxidase; GABA, γ -aminobutyric acid; met, methionine; SAM, *S*-adenosylmethionine; SAMDC, S-adenosylmethionine decarboxylase; dcSAM, decarboxylated *S*-adenosylmethionine; ACC, 1-aminocyclopropane-1-carboxylic-acid; ACC synthase, 1-aminocyclopropane-1-carboxylic-acid synthase.

Salt stress results in more and more production of stress ethylene content by enhancing the activity of ACC synthase enzyme, whereas, application of PUT showed a significant reduction in stress ethylene content (Figure 2). Application of PUT results in a reduction of activity of the ACC synthase enzyme that untimely leads to reduced ethylene production. Salinity stress results in electrolyte leakage and more of Na^+^ ion accumulation that leads to ion toxicity. However, the presence of PUT is found to be effective in reducing Na^+^ accumulation electrolyte leakage, and it will improve the Na^+^/K^+^ ratio. Also, a major change was seen in lycopene content, application of PUT helps in improving lycopene content in stressed plants (Figure 3(a–f)). In the presence of salinity, antioxidant enzymes such as SOD, CAT, and those included in the ascorbate-glutathione cycle, APX, and GR, significantly reduce oxidative damage.^64^ On PUT application, various antioxidant enzymes (SOD, CAT, APX, and GR) activity increased and helped in ROS detoxification mechanism under salinity stress (Figure 4(a–d)). The findings of this study showed that put raised APX and GR activities in the salt-stressed tomato leaves, and that was consistent with the findings of Zhao et al.^65^ A substantial amount of research has been done to learn more about the molecular processes that underlie plant’s reaction to abiotic stressors. Consequently, it has been determined that a number of upstream and downstream processes are linked to stress tolerance via protective mechanisms and metabolic connections, both of that PAs are essential components.^66^ Current findings provide clear molecular and biochemical evidence that PUT is linked to mitigate salinity in tomatoes. Evidence suggests that put is important for plant growth and development as well as for resistance response to the stress that impacts agricultural productivity. Applying competitive inhibitors, treating exogenous PAs, and using the most effective methods based on mutant plants are among the primary methods for examining the role of PA in plants.^24^ Recent findings shed light on PUT’s function in promoting plant development and defense toward stress. These findings will be reviewed to give a broad picture for future studies, and PUT may be used as a growth stimulant to improve resistance toward salinity in tomatoes (Figure 7).

## 5. Conclusion and future perspectives

Climatic change is significantly impacting agriculture due to various stresses of both biotics and abiotics, which is a major threat to global food security. Future agricultural productivity will drastically decrease, which will have a detrimental effect on the environment and the demand for food due to population growth. To achieve this ambitious demand for food security, we need to focus on developing crop varieties that can withstand various stresses. Salinity stress is one of the main hindrances in plant growth due to the excess accumulation of soluble ions (Na^+^, Ca^+^, K^+^, Mg^+^) in the root zone. Salt stress influences nearly half of the irrigated and 20% of the cultivated land worldwide. There is an urgent need to develop salt tolerant plant varieties for a sustainable approach to mitigate the global food production. In addition, a deeper understanding of tolerance mechanisms and the study of significant and dependable biochemical and physiological parameters related to salt tolerance are needed. In this regard, (PAs) and polyamine transporters (PUTs) are emerging as exciting areas of research because of their earlier reports on crucial roles in plant growth, stress tolerance, and overall resilience. While we have an understanding of how PAs function in many species, we still need to research more into this aspect of how PUTs specifically aid in plant growth and stress responses. Our results were found positive in tomato plants applying PUTs exogenously has been shown to boost growth and activate stress-related genes like ACS1, NHX1, HKT1;2, and SOS1 when faced with salinity stress. These genes play a role in removing the excess Na^+^, K^+^ accumulation in the root zone of the plant. Additionally, the PUTs enhance the activity of antioxidant enzymes (SOD, CAT, APX, and GR) and increase the level of ACC synthase and ethylene, helping to counteract the harmful effects of stress-induced ethylene. Under salt stress conditions, plants often face damage in the membrane integrity and increase electrolytic leakage (EL), which can be reduced by application of polyamines, similar results and decreases in EL and membrane stabilization were observed in tomato plants, indicating the role of membrane protection and leading to plant growth and crop productivity. Proline accumulation and polyamines are closely linked, especially during stress conditions, but the mechanism is not clearly understood, and it still needs to be investigated for further roles in protection from stress conditions and the physiology of the plants. SOD levels were found at higher concentrations in tomato plants with polyamine applications, which indicates their protective role of cellular components from oxidative damage and enhanced antioxidant enzymes provide stress tolerance. PUT treatment in tomato plants showed increased the shoot length with a greater number of leaves and a larger surface area providing a more photosynthetic area for plant productivity. Similarly, an increase in root length and less root damage has also been observed in our results, supporting the increase in biomass and enhanced nutrient uptake. This comprehensive morphological, physiological, and metabolic improvement under salinity stress underscores the potential of PUTs to enhance tomato resilience during cultivation and production.

Looking ahead, the adoption of PUTs as potent bio-stimulants in agricultural practices could revolutionize crop management and cultivation. By integrating PUTs into breeding programs and agricultural technologies, we can develop crops that are better equipped to withstand the stresses associated with severe climatic conditions. Our findings promote the application of polyamines as an effective practice to deal with salinity-induced stress in tomatoes and can also be used for other economically important crops. This approach has the potential to improve food security and promote sustainable agricultural practices, ultimately contributing to a more resilient global food system. Overall, the strategic use of PUTs represents a forward-thinking solution to some of the most pressing challenges in agriculture in various stresses, offering a pathway to a more sustainable and secure food future.

## Data Availability

I would like to declare that the work described is original research that has not been published previously, and is not under consideration for publication elsewhere, in whole or in part.
